# Focusing on the Apolipoprotein M–Mitophagy Axis: A Mechanism for Renal Protection in Diabetic Nephropathy

**DOI:** 10.1155/jdr/1498605

**Published:** 2026-03-19

**Authors:** Tianlei Chen, Min Yang

**Affiliations:** ^1^ Department of Nephrology, Changzhou First People’s Hospital, Changzhou, China

**Keywords:** apolipoprotein m, diabetic nephropathy, lipid metabolism, mitophagy, sphingosine-1-phosphate

## Abstract

Diabetic nephropathy (DN), a predominant cause of end‐stage renal disease (ESRD), is primarily driven bfigolic disturbances and mitochondrial dysfunction. Apolipoprotein M (ApoM), a protein associated with high‐density lipoprotein (HDL), is notably downregulated in DN and is correlated with a decline in renal function. Recent studies have identified a protective bidirectional axis between ApoM and mitophagy, the selective autophagy of mitochondria. ApoM, chiefly through its role as a carrier for sphingosine‐1‐phosphate (S1P), enhances mitophagy by activating the silent information regulator 1 (SIRT1) and parkin induced kinase 1 (PINK1)/Parkin pathways, thereby improving mitochondrial quality control. Conversely, mitophagy facilitates ApoM synthesis by supplying sufficient adenosine triphosphate (ATP) for its production and the assembly of HDL. In the context of DN, hyperglycemia disrupts this reciprocal relationship, leading to a detrimental cycle of impaired mitophagy and reduced ApoM, which exacerbates renal injury. Targeting the ApoM–mitophagy axis through ApoM enhancement or mitophagy activation emerges as a promising therapeutic approach for personalized renal protection in DN. This review synthesizes the mechanistic interplay between lipid metabolism and mitochondrial quality control, emphasizing its translational potential and the necessity for further investigation.

## 1. Introduction

Diabetic nephropathy (DN) is a severe complication of diabetes with high global burden, driven by multifactorial pathogenesis intertwined with metabolic disturbances [1]. Worldwide, 20%–40% of diabetic patients develop DN, which, once established, progresses irreversibly to end‐stage renal disease (ESRD), necessitating dialysis or transplantation and imposes significant healthcare and economic burdens [2]. The pathogenesis of DN involves complex interplay of multiple factors: chronic inflammation driven by pathways, such as nucleotide‐binding oligomerization domain, leucine rich repeat, and pyrin domain containing 3 (NLRP3) inflammasome activation and proinflammatory cytokine release [3]; renal fibrosis mediated by transforming growth factor‐beta (TGF‐β)/Smad signaling and epithelial‐mesenchymal transition [4]; epigenetic modifications including DNA methylation dysregulation of antioxidant and metabolic genes [5]; and gut microbiota dysbiosis that disrupts metabolite balance (e.g., reduced short‐chain fatty acids and elevated trimethylamine N‐oxide) [6]. Additionally, genetic polymorphisms contribute to individual susceptibility to DN progression [7].

Lipid metabolism disorder is a central and early driver of DN, closely intertwined with renal injury. In diabetic conditions, aberrant lipid accumulation in renal tubular epithelial cells and mesangial cells induces lipotoxicity, oxidative stress, and mitochondrial dysfunction [4, 8]. Proximal tubular epithelial cells, which rely heavily on fatty acid oxidation for energy, exhibit impaired lipid metabolism in DN, leading to triglyceride deposition, activation of proinflammatory pathways, and exacerbation of tubulointerstitial fibrosis [4]. Dysregulated lipid transport further amplifies renal damage by promoting macrophage infiltration and extracellular matrix deposition [4]. Notably, lipid metabolism disorders directly impair mitochondrial homeostasis, key for renal cell function, by disrupting fatty acid oxidation, inducing mitochondrial reactive oxygen species (ROS) production, and inhibiting mitochondrial quality control [8, 9]. This lipid‐induced mitochondrial dysfunction constitutes a critical pathological link between metabolic disturbances and renal injury in DN.

Mitochondrial dysfunction, particularly impaired mitophagy, is a core driver of DN, while apolipoprotein M (ApoM) emerges as a promising protective factor with under‐explored crosstalk with mitophagy. Impaired clearance of damaged mitochondria (mitophagy) leads to the accumulation of dysfunctional organelles, excessive ROS production, and subsequent renal cell apoptosis and fibrosis [10, 11]. Key mitophagy‐related proteins, including parkin induced kinase 1 (PINK1), parkin E3 ubiquitin ligase (Parkin), and prohibitin 2 (PHB2), are dysregulated in DN, further exacerbating mitochondrial homeostasis disruption [12–16]. Against this backdrop, ApoM, a 28‐kDa protein primarily associated with high‐density lipoprotein (HDL), has emerged as a promising protective factor [17]. ApoM is not only a key regulator of lipid metabolism (e.g., transporting sphingosine‐1‐phosphate [S1P]) but also exhibits anti‐inflammatory, antioxidant, and vasoprotective properties [2, 18]. Clinical studies show that serum ApoM levels are negatively correlated with DN severity: in patients with type 2 diabetes, lower ApoM levels predict a faster decline in estimated glomerular filtration rate (eGFR) over 5 years. In DN mouse models, ApoM deletion aggravates renal injury (increased proteinuria and renal fibrosis), while ApoM overexpression alleviates these phenotypes [2, 18–20].

Despite these independent findings, the link between ApoM and mitophagy in DN, particularly how ApoM‐mediated lipid metabolism regulation intersects with mitochondrial quality control, remains underexplored [21]. This gap is particularly critical in the context of interdisciplinary investigations into nutrition–metabolism–health interactions [22]: ApoM’s role in HDL lipid transport may directly influence mitophagy‐related energy homeostasis (e.g., via S1P‐mediated signaling or NAD + metabolism) [2, 23], yet few studies have addressed this crosstalk. This review aims to fill this gap by systematically exploring the ApoM–mitophagy axis in DN, emphasizing metabolic mechanisms [24], therapeutic potential [25, 26], and future research directions to advance personalized renal protection [27, 28].

### 1.1. Literature Search Strategy

To systematically synthesize evidence on the ApoM–mitophagy axis in DN, relevant literature was retrieved from PubMed, Web of Science, and Embase databases (from inception to August 2025) using key search terms: “apolipoprotein M” OR “apoM,” “mitophagy,” “diabetic nephropathy” OR “DN,” “sphingosine‐1‐phosphate” OR “S1P,” and “lipid metabolism.” Included studies focused on the regulatory crosstalk between ApoM and mitophagy in DN (encompassing human clinical research, animal models, and in vitro experiments) and included original articles, reviews, or meta‐analyses with rigorous design, while non‐English publications, case reports, conference abstracts, and studies unrelated to the ApoM–mitophagy axis or DN were excluded. Two authors independently screened titles, abstracts, and full texts with cross‐verification for discrepancies, prioritizing recent (2018–2025) high‐impact studies and landmark literature to ensure the timeliness and reliability of the synthesized evidence.

**Figure 1:**
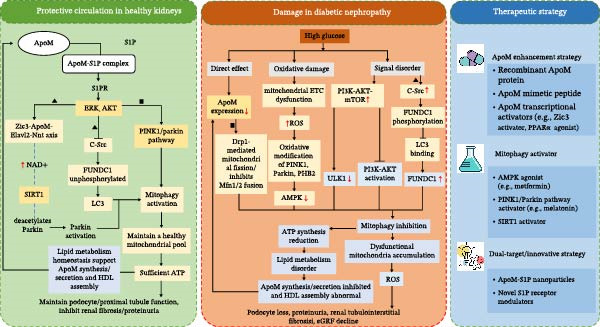
Schematic illustrating the bidirectional protective ApoM–mitophagy axis in renal health, its cell‐type‐specific disruption by diabetic hyperglycemia, and mechanism‐based therapeutic strategies. Key distinctions between experimentally validated and hypothetical links, as well as cell‐specific regulatory patterns, are indicated. Left panel (protective circulation in healthy kidneys): ApoM forms a complex with S1P and binds to S1PR, activating ERK/AKT signaling. This enhances mitophagy through two pathways. (1) Multiply validated pathway (black solid lines): AKT promotes SIRT1, and SIRT1 deacetylates Parkin to activate the PINK1/Parkin pathway; in podocytes (marked with △), ApoM–S1P inhibits c‐Src, preserving FUNDC1 (unphosphorylated at Tyr18) to mediate mitophagy. (2) Hypothetical pathway (blue dashed lines, animal/in vitro validation only): ERK directly phosphorylates PINK1 at Ser228 to enhance its kinase activity, and ApoM upregulates the Zic3–Elavl2–Nnt axis to increase NAD^+^ levels (supporting SIRT1 activity). Efficient mitophagy maintains a healthy mitochondrial pool, producing sufficient ATP and sustaining lipid metabolism, both essential for ApoM synthesis and HDL assembly, forming a reinforcing protective cycle. Central panel (damage in diabetic nephropathy): high glucose induces two interconnected insults. (1) Excessive ROS cause oxidative modification of PINK1/Parkin, and upregulate TIPE1 (in proximal tubular epithelial cells, marked with □) to promote ubiquitin‐dependent degradation of PHB2. (2) High glucose activates Drp1‐mediated mitochondrial fission (suppressing Mfn1/2 fusion) and dysregulates nutrient‐sensing pathways (AMPK inhibition and PI3K‐AKT‐mTOR activation), jointly inhibiting ULK1 and mitophagy initiation. In podocytes (△), high glucose activates c‐Src to phosphorylate FUNDC1 at Tyr18 (blocking LC3 binding). These disruptions reduce ApoM expression and impair mitophagy, leading to damaged mitochondria accumulation, ATP depletion, and a vicious cycle of renal injury (e.g., podocyte loss and tubular fibrosis). Right panel (therapeutic strategy): interventions targeting the ApoM–mitophagy axis are categorized by mechanism. (1) ApoM enhancement (black solid lines for animal validation and blue dashed lines for in vitro only): recombinant ApoM protein/ApoM–S1P complexes restore S1PR signaling; Zic3 activators/PPARα agonists (e.g., fenofibrate) upregulate ApoM to reactivate the Zic3–Elavl2–Nnt axis. (2) Mitophagy activation (black solid lines for preclinical validation): AMPK agonists (e.g., metformin) activate ULK1/PINK1; PINK1/Parkin pathway activators (e.g., melatonin) promote mitophagic flux. (3) Dual–target strategies (blue dashed lines and preclinical exploration): ApoM–S1P nanoparticles target renal cells via SR‐BI receptors; novel S1P analogs co‐activate S1PR and AMPK to synergistically restore the axis. Thin red upward/downward arrows indicate upregulation/downregulation; black solid lines indicate multiply validated pathways (human clinical + animal + in vitro); blue dashed lines indicate hypothetical pathways (animal/in vitro validation only); △ indicates podocyte‐specific pathway; and □ indicates proximal tubular epithelial cell‐specific pathway. AMPK, AMP‐activated protein kinase; ApoM, apolipoprotein M; Drp1, dynamin‐related protein 1; FUNDC1, FUN14 domain containing 1; mTOR, mammalian target of rapamycin; PHB2, prohibitin 2; ROS, reactive oxygen species; S1P, sphingosine‐1‐phosphate; S1PR, S1P receptor; SIRT1, silent information regulator 1; TIPE1, tumor necrosis factor alpha‐induced protein 8‐like 1; ULK1, unc‐51 like autophagy activating kinase 1.

Figure 1 describes the ApoM–mitophagy protective axis and its therapeutic targeting in DN.

## 2. Mitophagy: A Critical Player in DN

Mitophagy is a highly conserved cellular process that selectively removes damaged or dysfunctional mitochondria, maintaining the quality and quantity of the mitochondrial pool [15]. In renal tissues, this process is indispensable for preserving the function of tubular epithelial cells and mesangial cells, both of which rely heavily on mitochondrial adenosine triphosphate (ATP) production for physiological activities [18]. In DN, mitophagy is severely impaired, and this dysfunction serves as a key pathological link between high glucose exposure and renal injury [14, 29–31].

### 2.1. Mechanisms of Mitophagy Dysregulation in DN

Mitophagy dysregulation in DN is mediated by disrupted physiological pathways and high glucose‐induced metabolic perturbations. Under physiological conditions, mitophagy is primarily regulated through two well‐established mechanisms: the PINK1/Parkin‐dependent pathway and receptor‐mediated pathways such as those involving FUN14 Domain Containing 1 (FUNDC1), BCL2 interacting protein 3 (Bnip3), and Nix. The PINK1/Parkin pathway is particularly well‐characterized: upon mitochondrial damage and loss of membrane potential, PINK1 accumulates on the outer mitochondrial membrane where it undergoes autophosphorylation and phosphorylates ubiquitin. This prompts the recruitment of Parkin, an E3 ubiquitin ligase, which ubiquitinates outer membrane proteins such as voltage‐dependent anion channel 1 (VDAC1) and PHB2. These ubiquitinated proteins act as recognition signals for LC3‐decorated autophagosomes, leading to the engulfment and subsequent lysosomal degradation of damaged mitochondria [15, 32, 33]. Receptor‐mediated pathways, including that mediated by the outer membrane protein FUNDC1 under hypoxia, provide an alternative route by directly engaging LC3 to facilitate mitophagy [12, 14].

In DN, chronic exposure to high glucose disrupts these protective mitophagic processes through multiple interrelated mechanisms. Excessive ROS generated by mitochondrial electron transport chain complexes under high glucose conditions cause oxidative modifications to key mitophagic proteins, such as PINK1 and Parkin, impairing their function and initiating a vicious cycle of mitochondrial damage and defective clearance. Concurrently, high glucose alters mitochondrial dynamics by promoting Drp1‐mediated fission and suppressing fusion proteins, like Mfn1 and Mfn2, resulting in small, dysfunctional mitochondrial fragments that evade autophagic recognition and compromise organelle repair [11, 34, 35].

Furthermore, high glucose modulates major nutrient‐sensing pathways that regulate mitophagy. AMP‐activated protein kinase (AMPK) activity is suppressed due to disrupted AMP/ATP ratios and ROS‐mediated inhibition, reducing phosphorylation of its downstream target unc‐51 like autophagy activating kinase 1 (ULK1) and impairing mitophagic initiation. At the same time, high glucose activates the phosphatidylinositol 3‐kinase (PI3K)‐Akt‐mammalian target of rapamycin (mTOR) pathway, which further inhibits ULK1 and suppresses mitophagy [36, 37]. Together, these alterations create a synergistic inhibitory effect on mitophagic flux, contributing significantly to the progression of DN.

Notably, mitophagy dysregulation exhibits cell‐type specificity: podocytes primarily rely on the FUNDC1‐mediated hypoxic mitophagy pathway [12], and high glucose‐induced c‐Src activation preferentially inhibits FUNDC1 function in these cells, leading to podocyte loss and proteinuria [12]. In contrast, proximal tubular epithelial cells depend more on the PINK1/Parkin pathway [18], where high glucose‐induced ROS directly impairs PINK1/Parkin stability, exacerbating tubular lipid deposition and fibrosis [38].

### 2.2. Dysregulation of Mitophagy‐Related Proteins in DN

Dysregulation of key mitophagy‐related proteins further exacerbates mitochondrial homeostasis disruption in DN. For instance, tumor necrosis factor alpha‐induced protein 8‐like 1 (TIPE1) is markedly upregulated in renal tubular epithelial cells in both DN patients and animal models [13]. TIPE1 directly binds to the mitophagy receptor PHB2 and promotes its ubiquitination and proteasomal degradation, thereby impairing LC3‐mediated engulfment of damaged mitochondria [13], and clinically, reduced PHB2 levels are associated with increased renal fibrosis and decreased eGFR in DN patients [13]. Additionally, ULK1, a serine/threonine kinase essential for autophagy initiation, is downregulated in diabetic renal tissues, and its expression correlates inversely with the severity of renal injury [39, 40]. Restoration of ULK1 expression in DN models has been shown to enhance mitophagic flux, reduce ROS production, and attenuate fibrosis, underscoring its central role in maintaining mitochondrial quality control [39, 41, 42]. Another key pathway involves FUNDC1, which mediates hypoxia‐induced mitophagy, a process particularly relevant in DN where microvascular injury causes renal hypoxia [43–45]. In diabetic conditions, activation of the tyrosine kinase c‐Src leads to phosphorylation of FUNDC1 at tyrosine 18, inhibiting its interaction with LC3 and suppressing mitophagy [12], while pharmacological inhibition of c‐Src rescues FUNDC1 function, promotes mitophagy, and ameliorates renal damage in experimental DN [12].

### 2.3. Mitophagy‐Targeted Interventions in DN

Several agents have shown preclinical efficacy in restoring mitophagy and alleviating DN through distinct mechanisms [46, 47]. Melatonin, a pineal hormone known for its antioxidant and circadian regulatory functions, promotes mitophagy in DN by activating the AMPK–PINK1 pathway [46]. It enhances AMPK phosphorylation, facilitating PINK1 translocation to damaged mitochondria and subsequent Parkin recruitment, thereby increasing mitophagic flux [48, 49]. The renal protective effects of melatonin are abolished upon PINK1 knockdown or AMPK inhibition, confirming the dependence on this pathway. Additionally, by clearing dysfunctional mitochondria, melatonin attenuates oxidative stress and mitigates renal injury [46]. Similarly, erythropoietin (EPO), widely recognized for its role in erythropoiesis, exerts renal protection in DN by enhancing PINK1/Parkin‐mediated mitophagy. EPO upregulates PINK1 and Parkin expression, promotes LC3‐mitochondria colocalization, and increases autophagosome formation [47, 50]. It also improves mitochondrial health by reducing fragmentation, restoring membrane potential, and decreasing ROS production [51, 52]. These benefits are nullified by PINK1 knockdown, underscoring the essential role of the PINK1/Parkin pathway. In vivo, EPO treatment reduces proteinuria, fibrosis, and oxidative stress in diabetic mice while elevating renal PINK1 and Parkin levels [47].

In contrast, *Angelica sinensis* polysaccharide, a bioactive compound with anti‐inflammatory and antioxidant properties, appears to modulate mitophagy through a different mechanism. In DN models, it suppresses excessive mitophagy by reducing the LC3II/LC3I ratio and Nix expression while increasing p62, indicating inhibited autophagic flux. It also downregulates Drp1, attenuating mitochondrial fission and promoting structural integrity. These effects are accompanied by reduced AMPK and mTOR expression at both protein and mRNA levels, suggesting a balancing role in mitophagy regulation that ultimately delays DN progression [53].

## 3. ApoM: Biological Roles and Interaction With Mitophagy in DN

ApoM is a unique component of HDL with diverse biological functions, ranging from lipid metabolism regulation to cellular signaling modulation [54]. In recent years, accumulating evidence has highlighted its critical role in DN, where it acts as a protective factor by integrating lipid metabolic pathways with mitochondrial quality control, specifically mitophagy [2, 55]. This section details the biological characteristics of ApoM, its protective mechanisms in DN, and its bidirectional interaction with mitophagy.

### 3.1. Structural and Metabolic Characteristics of ApoM

ApoM is a 254‐amino acid protein with a relative molecular mass of approximately 28 kDa, encoded by the APOM gene located on chromosome 6q27 in humans. It is primarily synthesized in the liver and kidney (especially proximal tubular epithelial cells) and is predominantly associated with HDL (accounting for ~5%–10% of HDL proteins), though small amounts are also found in chylomicrons and very low‐density lipoproteins. The structural features of ApoM are critical to its function: it contains an N‐terminal signal peptide (responsible for secretion) and a C‐terminal mature peptide (the functional domain), which includes a lipocalin fold, a conserved structure that binds small hydrophobic ligands, most notably S1P [2].

ApoM exhibits high evolutionary conservation across species, reflecting its essential biological functions. Initially recognized as a regulator of lipid metabolism, ApoM plays a key role in HDL maturation and S1P transport: it binds S1P with high affinity, protecting S1P from degradation and mediating its delivery to target cells via HDL. This transport function is critical, as S1P is a multifunctional lipid mediator that regulates cell survival, proliferation, migration, and angiogenesis via its 5 g protein‐coupled receptors (S1P1–S1P5) [2, 56]. Beyond lipid metabolism, ApoM exhibits anti‐inflammatory, antioxidant, and antithrombotic properties, all of which contribute to its protective role in vascular and renal diseases [2]. Under physiological conditions, ApoM is predominantly expressed in proximal tubular epithelial cells and, to a lesser extent, in glomerular endothelial cells and mesangial cells of the kidney [57, 58]. However, in diabetic states, its expression is selectively downregulated in proximal tubular epithelial cells, consistent with the cell type’s high reliance on fatty acid oxidation and susceptibility to lipotoxicity [11, 59], while glomerular endothelial cell ApoM levels remain relatively preserved, suggesting a cell‐specific regulatory pattern in DN.

### 3.2. Protective Mechanisms of ApoM in DN

ApoM exerts renal protection in DN through interconnected pathways leveraging its lipid transport and signaling functions [2]. ApoM binds S1P and facilitates its delivery to renal cells, where activation of the S1P1 receptor inhibits the TGF‐β/Smad3 fibrotic signaling pathway. Specifically, S1P1 triggers PI3K‐Akt signaling [60], leading to Akt‐mediated phosphorylation of Smad3 at serine 208 and preventing its nuclear translocation [61, 62]. This results in reduced transcription of profibrotic genes such as collagen I and fibronectin [63]. In vivo, ApoM deletion exacerbates renal fibrosis, whereas ApoM overexpression or S1P supplementation attenuates Smad3 activation and fibrotic injury [2]. Concurrently, the ApoM/S1P axis improves renal hemodynamics by enhancing endothelial nitric oxide synthase (eNOS) activity. Through S1P1‐mediated activation of Akt and extracellular signal‐regulated kinase (ERK) pathways, ApoM promotes eNOS phosphorylation at serine 1177, increasing nitric oxide production and supporting vasodilation. In DN, diminished ApoM levels correlate with impaired eNOS function, glomerular hyperfiltration, and proteinuria. Restoration of ApoM normalizes renal blood flow and reduces glomerular pressure in diabetic models [2, 64].

Furthermore, ApoM contributes to mitochondrial protection via upregulation of silent information regulator 1 (SIRT1), a mechanism primarily supported by in vitro cellular experiments (e.g., human proximal tubular epithelial HK‐2 cells) and murine DN models. By activating the S1P1/ERK pathway, ApoM enhances forkhead box o3a‐driven SIRT1 transcription and helps maintain NAD^+^ levels essential for SIRT1 activity. Increased SIRT1 deacetylates downstream targets, including peroxisome proliferator‐activated receptor gamma coactivator 1‐alpha and Parkin, improving mitochondrial biogenesis and mitophagy [65]. Notably, direct evidence for this regulatory cascade in human renal tissues remains limited, though clinical studies have correlated lower serum ApoM levels with reduced SIRT1 expression in DN patients [66]. ApoM deficiency is associated with reduced SIRT1 expression and aggravated mitochondrial dysfunction in mice, while its overexpression preserves mitochondrial integrity in murine DN models [67].

### 3.3. Bidirectional Interaction Between ApoM and Mitophagy

ApoM and mitophagy form a bidirectional regulatory axis that maintains renal metabolic and mitochondrial homeostasis. Cumulative evidence from in vitro (podocytes and human proximal tubular epithelial HK‐2 cells) and murine DN models [12, 68] confirms ApoM acts as an upstream regulator of mitophagy, with this regulatory direction consistent across key renal cell types. Notably, this regulation is context‐dependent—its efficacy is attenuated in advanced DN characterized by severe lipotoxicity or excessive ROS, which impair ApoM’s S1P‐binding capacity and downstream signaling [8, 31]. ApoM modulates mitophagy through several key mechanisms rooted in its metabolic roles. It influences the Zic3–ApoM–Elavl2–N‐methyltransferase (Nnt) axis, wherein the transcription factor Zic3 promotes ApoM transcription, and ApoM enhances cytoplasmic translocation of the RNA‐binding protein Elavl2. Elavl2 stabilizes Nnt mRNA, increasing nicotinamide Nnt expression and activity. This elevates intracellular NAD^+^ levels, thereby activating the NAD^+^‐dependent deacetylase SIRT1, which promotes mitophagy through deacetylation of targets such as Parkin [68–70]. Additionally, ApoM, via its carrier role for S1P, activates the S1P1 receptor and subsequent ERK signaling, which phosphorylates PINK1 at serine 228 to enhance its kinase activity [66, 71], this phosphorylation event and its downstream promotion of PINK1/Parkin‐mediated mitophagy are currently supported by in vitro studies using podocytes and proximal tubular epithelial cells [67, 72]. Direct evidence for ERK–PINK1 crosstalk in human renal tissues is lacking, and human PINK1 phosphorylation sites and regulatory kinetics may differ from those in murine cells [73].

ApoM also exerts antioxidant effects by reducing ROS production and enhancing scavenging mechanisms [74, 75]. By mitigating oxidative stress, ApoM preserves the functionality of mitophagy‐related proteins, such as PINK1 and Parkin, and helps maintain mitochondrial membrane potential, ensuring targeted removal of damaged organelles without unnecessary initiation of mitophagy under mild stress [2]. Cell‐specific vulnerability to ApoM loss further highlights the axis’s relevance. ApoM deficiency predominantly aggravates podocyte injury by reducing S1P‐mediated FUNDC1 activation [12], while in proximal tubular epithelial cells, it impairs ATP production via compromised PINK1/Parkin mitophagy, amplifying tubulointerstitial fibrosis [8]. This cell‐type‐specific dependence underscores the need for tailored interventions targeting the ApoM–mitophagy axis in distinct renal cells.

Mitophagy plays an essential role in sustaining ApoM synthesis and functionality. Notably, current evidence does not support direct transcriptional regulation of ApoM by mitophagy‐related factors (e.g., PINK1 and Parkin); instead, mitophagy indirectly maintains ApoM’s synthesis and HDL incorporation by preserving mitochondrial ATP production and lipid metabolism homeostasis [18, 76]. As a secreted protein, ApoM requires ATP for its synthesis and secretion, processes heavily reliant on mitochondrial ATP production. Impaired mitophagy in DN diminishes ATP availability, thereby reducing ApoM expression [68, 77]. Moreover, mitophagy supports mitochondrial contribution to HDL assembly by providing essential lipids and sustaining ATP‐dependent transporters such as ATP‐binding cassette transporter A1 [78]. Preserved mitophagy ensures adequate incorporation of ApoM into HDL particles, which enhances its stability and S1P‐binding capacity, thereby maintaining its bioactivity [79]. This reciprocal maintenance between ApoM and mitophagy underscores a metabolically coordinated loop that is disrupted in DN and represents a potential target for therapeutic intervention.

## 4. Therapeutic Potential of Targeting the ApoM–Mitophagy Axis in DN

The ApoM–mitophagy axis has emerged as a promising metabolism‐oriented target for DN, with significant potential for both biomarker‐based stratification and therapeutic intervention.

### 4.1. ApoM as a Type‐Specific Biomarker

ApoM demonstrates considerable prognostic value in DN, though its behavior differs between diabetes types [28, 80]. In type 2 diabetes, lower serum ApoM levels are associated with more severe renal impairment. A 5‐year prospective study found that patients with ApoM levels ≤1.2 mg/dL had a 2.3‐fold increased risk of significant eGFR decline. Low ApoM correlates with elevated urinary albumin‐to‐creatinine ratio and profibrotic markers such as collagen I and TGF‐β [2]. Conversely, in type 1 diabetes, higher ApoM levels predict adverse renal outcomes. Data from the diabetes control and complications trial (DCCT)/epidemiology of diabetes interventions and complications (EDIC) cohort showed that each 0.1 mg/dL increase in ApoM was linked to a 15% greater risk of progressive proteinuria and chronic kidney disease [28]. This type‐specific discrepancy underscores the importance of population‐specific biomarker validation. Combining ApoM with mitophagy‐related markers, such as serum PINK1 or urinary PHB2, may enhance prognostic accuracy and support personalized risk assessment [13, 81].

### 4.2. Therapeutic Strategies to Target the Axis

Several mechanism‐informed strategies are under preclinical evaluation to restore ApoM–mitophagy signaling. ApoM enhancement approaches include the use of recombinant ApoM protein or ApoM–S1P complexes, which have been shown to reduce proteinuria and fibrosis in streptozotocin‐induced or db/db murine DN models by promoting S1P signaling and mitophagy [2]. Alternatively, ApoM mimetic peptides that mimic its S1P‐binding domain offer oral bioavailability and lower immunogenicity, though their efficacy has only been validated in in vitro cell models [82]. It is important to note that these strategies have not yet entered human clinical trials [83], and their translational potential is limited by species‐specific differences in ApoM function and HDL metabolism [84, 85]. Meanwhile, transcriptional activation of ApoM, via Zic3 activators or peroxisome proliferator, activated receptor alpha agonists, such as fenofibrate, can elevate ApoM expression and reactivate downstream pathways, such as Zic3–Elavl2‐Nnt, improving mitophagy and renal outcomes [68].

Mitophagy activation provides a complementary strategy. AMPK–PINK1 pathway agonists including melatonin and metformin enhance mitophagic flux, decrease fibrosis, and support ATP‐dependent ApoM synthesis [46]. Direct PINK1 or Parkin activators, such as the small molecule PIK‐75, also show efficacy in reducing mitochondrial ROS and improving mitophagy in experimental DN [68]. Dual‐target interventions simultaneously engage both arms of the axis. HDL‐mimetic nanoparticles co‐loaded with ApoM and mitophagy activators, such as melatonin, enable targeted delivery to renal cells via SR‐BI receptors, synergistically increasing ApoM levels and mitophagy activity while reducing proteinuria [77]. Similarly, novel S1P analogs that activate both S1P1 signaling and AMPK can mimic ApoM’s protective effects while directly promoting mitophagy [18]. These approaches highlight the clinical viability of the ApoM–mitophagy axis and emphasize its role in bridging metabolic regulation with mitochondrial quality control, an alignment that may ultimately support more effective and personalized treatments for DN.

### 4.3. Translational Barriers and Considerations

Despite promising preclinical evidence, translating ApoM–mitophagy axis‐targeted therapies to human DN faces notable species‐specific and pathological barriers. First, species differences in ApoM expression: human ApoM is mostly synthesized in the liver (≈70%) and renal proximal tubular cells (≈25%), while murine kidneys express a higher proportion (≈40%) relative to the liver [86], potentially altering intervention efficacy. Second, HDL metabolism divergence: ApoM accounts for 5%–10% of human HDL proteins versus 12%–15% in murine HDL [83], with human HDL carrying less S1P per particle, weakening ApoM–S1P signaling [87]. Third, pathological heterogeneity: preclinical models (e.g., streptozotocin‐induced diabetes) focus on hyperglycemia‐driven injury, while human DN involves comorbidities (hypertension and aging) and genetic polymorphisms that modify axis function [88]. Finally, limited human renal tissue evidence: key mechanisms (e.g., ERK–PINK1 phosphorylation and SIRT1–Parkin crosstalk) lack validation in human biopsies, and serum ApoM may not reflect renal tissue‐specific function [18, 47]. These barriers underscore the need for human‐centric studies (renal biopsy‐based analyses and phase I trials) to bridge preclinical and clinical translation.

## 5. Future Directions and Clinical Urgency

Despite significant gaps in understanding the ApoM–mitophagy axis in DN, its clinical translation is urgently needed, current treatments only slow rather than halt or reverse disease progression, with a substantial proportion of patients progressing to ESRD [89]. The global burden of DN underscores this urgency: axis dysregulation occurs early, 3–5 years before overt proteinuria, offering a critical window for preventive strategies that could drastically reduce ESRD‐related healthcare burdens. Resolving unresolved mechanistic questions, such as the specific interaction of ApoM with mitophagy receptors FUNDC1 [12, 90] and PHB2 [13], is foundational to advancing targeted therapies.

Priority research should focus on actionable, evidence‐based goals rooted in existing findings. First, validate ApoM as a type‐specific prognostic biomarker across diverse populations, combining it with mitophagy‐related markers (serum PINK1 and urinary PHB2) to enhance predictive accuracy [91]. Second, deepen mechanistic insights using advanced techniques: employ co‐immunoprecipitation–mass spectrometry to map ApoM’s binding with mitophagy regulators, and single‐cell sequencing to clarify cell‐type‐specific axis function in renal tissues. Third, advance preclinical‐to‐clinical translation of interventions: test ApoM mimetic peptides or Zic3 activators for ApoM enhancement [68], and AMPK/PINK1 pathway [46] agonists (e.g., melatonin and metformin) for mitophagy activation, with defined endpoints such as reduced proteinuria and improved mitochondrial function.

Translational efforts must also prioritize precision and human relevance to bridge basic science and clinical practice. Utilize induced pluripotent stem cell‐derived renal organoids from DN patients with APOM polymorphisms to screen candidate drugs, ensuring preclinical findings align with human physiology. Develop HDL‐mimetic nanoparticles for targeted delivery of ApoM and mitophagy activators, minimizing off‐target effects while enhancing renal bioavailability. These steps will enable personalized strategies, such as ApoM supplementation for type 2 DN or targeted mitophagy activation for type 1 DN, that overcome the limitations of conventional therapies. Accelerating these research priorities is critical to capitalize on the reversible nature of early axis dysregulation, ultimately improving patient outcomes worldwide.

## 6. Conclusion

This review examines the ApoM–mitophagy axis as a key mechanism linking lipid metabolism and mitochondrial quality control in DN. ApoM, an HDL associated protein, promotes mitophagy through S1P mediated lipid signaling and NAD + metabolism, while mitophagy in turn supports ApoM synthesis by maintaining mitochondrial ATP production and HDL assembly. In DN, high glucose disrupts this reciprocal relationship, leading to a vicious cycle of impaired mitophagy and reduced ApoM that accelerates renal injury. Targeting this axis offers promising therapeutic strategies, such as ApoM enhancement or mitophagy activation, for personalized renal protection. Despite remaining mechanistic and clinical validation challenges, advancing research on the ApoM–mitophagy axis may translate into effective therapies that prevent or reverse DN progression, significantly improving patient outcomes worldwide.

## Author Contributions

**Tianlei Chen:** conceptualization, methodology, investigation, data curation, writing – original draft. **Min Yang:** supervision, project administration, writing – review and editing, funding acquisition.

## Funding

The authors sincerely acknowledge the financial support from the Beijing Bethune Charitable Foundation (Grant SCWLKY‐018) and the Changzhou City Applied Basic Research Program (Grant CJ20253090).

## Disclosure

Both authors provided final approval of the version to be published and agreed to be accountable for all aspects of the work.

## Ethics Statement

The authors have nothing to report.

## Conflicts of Interest

The authors declare no conflicts of interest.

## Data Availability

This is a review of the literature, and no new datasets were generated or analyzed during the current study. Data sharing is not applicable to this article.
